# Genome-Wide Association Study of Parity in Bangladeshi Women

**DOI:** 10.1371/journal.pone.0118488

**Published:** 2015-03-05

**Authors:** Briseis Aschebrook-Kilfoy, Maria Argos, Brandon L. Pierce, Lin Tong, Farzana Jasmine, Shantanu Roy, Faruque Parvez, Alauddin Ahmed, Tariqul Islam, Muhammad G. Kibriya, Habibul Ahsan

**Affiliations:** 1 Department of Health Studies, The University of Chicago, Chicago, Illinois, United States of America; 2 Comprehensive Cancer Center, The University of Chicago, Chicago, Illinois, United States of America; 3 UChicago Research Bangladesh, Ltd., Dhaka, Bangladesh; 4 Department of Environmental Health Sciences, Mailman School of Public Health, Columbia University, New York, New York, United States of America; 5 Departments of Medicine and Human Genetics, The University of Chicago, Chicago, Illinois, United States of America; David Geffen School of Medicine at UCLA, UNITED STATES

## Abstract

Human fertility is a complex trait determined by gene-environment interactions in which genetic factors represent a significant component. To better understand inter-individual variability in fertility, we performed one of the first genome-wide association studies (GWAS) of common fertility phenotypes, lifetime number of pregnancies and number of children in a developing country population. The fertility phenotype data and DNA samples were obtained at baseline recruitment from individuals participating in a large prospective cohort study in Bangladesh. GWAS analyses of fertility phenotypes were conducted among 1,686 married women. One SNP on chromosome 4 was non-significantly associated with number of children at P <10^-7^ and number of pregnancies at P <10^-6^. This SNP is located in a region without a gene within 1 Mb. One SNP on chromosome 6 was non-significantly associated with extreme number of children at P <10^-6^. The closest gene to this SNP is *HDGFL1*, a hepatoma-derived growth factor. When we excluded hormonal contraceptive users, a SNP on chromosome 5 was non-significantly associated at P <10^-5^ for number of children and number of pregnancies. This SNP is located near *C5orf64*, an open reading frame, and *ZSWIM6*, a zinc ion binding gene. We also estimated the heritability of these phenotypes from our genotype data using GCTA (Genome-wide Complex Trait Analysis) for number of children (h_g_^2^ = 0.149, SE = 0.24, p-value = 0.265) and number of pregnancies (h_g_^2^ = 0.007, SE = 0.22, p-value = 0.487). Our genome-wide association study and heritability estimates of number of pregnancies and number of children in Bangladesh did not confer strong evidence of common variants for parity variation. However, our results suggest that future studies may want to consider the role of 3 notable SNPs in their analysis.

## Introduction

Fertility i.e., the number of children that a woman will have in her life, is a relevant aspect of health, for individual family planning, clinical care, and for understanding long-term population growth. Challenges to fertility can arise from genetic abnormalities, in addition to infectious or environmental agents, delayed childbearing, behavior, and certain diseases [1]. Relative to the birth rate in developed countries, the fertility rate in Bangladesh has been historically high, largely because of a pattern of early childbearing and socioeconomic factors [2–7]. Following a decline in fertility in Bangladesh in the late 1970s and 1980s from 6.3 to 3.4 births per woman, the fertility rate plateaued at 3.3 for about ten years during the 1990s and then resumed its decline during the early 2000s [2–7]. According to the 2011 Bangladesh Demographic and Health Survey, the total fertility rate for Bangladeshi women aged 15–49 years is 2.7 births per woman, including a rate of 2.4 in urban areas and 2.7 in rural areas.

Human fertility is a complex trait determined by gene-environment interactions in which genetic factors represent a significant component [8–9]. Studies on fertility candidate genes have identified alleles involved in the occurrence of reproductive system diseases causing infertility or subfertility [10–13]. For example, in recent years, a new physiological role in human fertility regulation has emerged for the tumor-suppressor *p53 gene (P53)* as the *P53* Arg72Pro polymorphism has been associated with recurrent implantation failure in humans [14]. However, a significant proportion of human infertility remains unexplained. The emerging evidence indicates that fertility genes represent a set of genes whose role is changing, and acquiring clinical relevance, possibly due to their interaction with the prevalent environmental context, such as changing reproductive patterns (e.g., birth control, family planning, delayed childbearing, and spacing birth order). Although some polymorphic variants (alleles) have been identified in fertility genes, how these alleles affect human fertility and interact with the reproductive environment remains unclear [15–16].

To better understand inter-individual variability in fertility, we performed one of the first genome-wide association study (GWAS) of common female fertility phenotypes in humans in a large rural population of married Bangladeshi women who were not selected for impaired fertility. The study population has not altered their reproductive behavior as the western women. Although family planning campaigns have been implemented in rural Bangladesh the average fertility is still significantly higher in this population than in middle- and high-income countries.

## Materials and Methods

### Study description

The DNA samples genotyped in this study were obtained at baseline recruitment from individuals participating in the Health Effects of Arsenic Longitudinal Study (HEALS) [17]. GWAS analyses of fertility phenotypes were conducted using questionnaire responses and single nucleotide polymorphism (SNP) data on 1,691 women randomly selected from the HEALS study. HEALS [17] is a prospective investigation of health outcomes associated with arsenic exposure through drinking water in a cohort of adults in Araihazar, Bangladesh, a rural area southeast of the capital city, Dhaka. Between October 2000 and May 2002, we recruited healthy married individuals (age 18–75 years) who were residents of the study area for at least five years and primarily consumed drinking water from a local well. We enumerated 65,876 individuals residing in Araihazar, from which using a population-based sampling frame, 11,746 men and women were enrolled. During 2006–2008, additional recruitment of 8,287 participants using the same methodologies from the same underlying source population expanded the cohort size to over 20,000 individuals. A subset of women married 2 years or longer in the cohort with GWAS data were included in this analysis (N = 1,686).

At baseline, trained study physicians conducted in-person interviews and clinical evaluations and collected spot urine and blood samples from participants in their homes using structured protocols. The questionnaire collected extensive information on demographic and lifestyle factors [17]. This included age, television ownership, medication use (including hormonal contraception use), smoking status, study stage, and enrollment year. Arsenic exposure was also assessed in the study population using two primary measures, drinking water and urinary arsenic concentrations [17].

The study protocol was approved by the Institutional Review Boards of The University of Chicago, Columbia University, and the Bangladesh Medical Research Council. Informed consent was obtained from all participants. Because of the prevalence of illiteracy in our study population, consent was obtained verbally and documented by study physician. The consent procedure was approved by the three Institutional Review Boards who provide oversight for this project.

### Fertility phenotypes

The fertility phenotypes, including number of children and number of pregnancies, were measured during the baseline in-person interviews. Only women were asked to report on the number of living children and the number of pregnancies. The number of pregnancies variable included deaths, abortions and stillbirths, whereas the children variable only reflected number of live children. Although correlated at 0.99, both fertility phenotypes were included in the analysis as they could measure different fertility associated issues. We created an additional variable, ≥6 versus ≤1 children, to assess the extremes of fertility from the interview data. Hormonal contraception reflects current use of any type (oral, injectable, etc.) of hormonal contraception.

### Genotyping and quality control

DNA was extracted from clot blood using Flexigene DNA kit (Cat # 51204) from Qiagen. Concentration and quality of all extracted DNA were checked by Nanodrop 1000. As starting material, 250 ng of DNA was used and genotyping was conducted using Illumina HumanCytoSNP- 12 v2.1 chips with 299,140 markers. Standard Illumina protocol was used for scanning the chips on the BeadArray Reader and processing the image data in BeadStudio software to generate genotype calls.

Standard GWAS QC was conducted among the 299,140 SNPs. SNPs were excluded with (1) poor call rates (<95%), (2) monomorphic SNPS, and (3) HWE p-values <10^–10^. This QC resulted in individuals with high-quality genotype data for 259,597 SNPs. Imputation was performed using MaCH on the basis of the HapMap 3 Gujarati Indians in Houston (GIH) population (Build 36). We also implemented the following QC exclusion criteria for SNPs post-imputation: (1) MAF <0.005 and (2) SNP imputation score <0.3. Both genotyped and imputed SNPs were included in these analyses, which yielded 1,211,988 million SNPs after QC procedures. All QC was performed using PLINK [18].

### Statistical methods

Despite population-based sampling, the study population, being stable with geography-defined residence, included some related individuals. Standard GWAS analysis methods are not appropriate for related individuals due to lack of independence of genotypes. The EIGENSTRAT program revealed that all subjects in this Bangladeshi sample were clustered together and could not be assigned into subgroups, indicating that there was no significant population stratification within the sample [19]. EMMAX (Efficient Mixed Model Association Expedited) was also used to account for cryptic relatedness. EMMAX was originally developed to account for known relationships among highly inbred mouse strains, has recently been shown to reliably account for related sample structure, including relationships among cases [20]. EMMAX uses a variance component method that utilizes an empirically estimated relatedness matrix to model the correlation between genotypes of all cases and controls [19]. EMMAX, therefore, supplies the statistical control necessary to remove excess inflation in test statistics due to the known non-independence of cases and the possible non-independence of controls. EMMAX performs a linear mixed model regression for each marker that includes the previously estimated relatedness matrix as a covariate. EMMAX reports the Armitage trend test score for each marker and associated p value [21]. We consider SNPs to be genome-wide significant if the significance exceeds a P-value of 5 × 10^−8^ [22]. However, we present findings for SNPs that reached P <10^–5^ to consider whether our strongest associations are consistent with previous studies [23]. We show QQ plots of the p values resulting from the analysis of all SNPs in an EMMAX transformed distribution of Χ_1_^2^ random variables plotted against 1 million randomly generated Χ_1_^2^ observations. The QQ plot demonstrates close agreement between the two distributions, indicating that population stratification and cryptic relatedness are adequately controlled. Furthermore, the inflation parameter calculated with the transformed p values and the randomly generated observations is 1.03, which is also within the generally accepted limit (i.e. less than 1.05) [24].

The final models were adjusted for age, study stage, TV ownership, hormonal contraceptive use, enrollment year, in addition to being adjusted for relatedness using the EMMAX procedure described above. We did not adjust for urinary arsenic exposure or smoking status in our final models as adjustment did not result in a substantive change. We also conducted a sensitivity analysis, excluding women who report current use of hormonal contraceptives. The models exclduing hormonal contraceptive users were adjusted for age, study stage, TV ownership, enrollment year, and relatedness.

Regional association plots were created using the locus zoom “plot your own data” function (https://statgen.sph.umich.edu/locuszoom/genform.php?type=yourdata). Plots were created utilizing the genome build/LD population hg19/1000 Genomes Mar 2012 EUR.

For the number of children and pregnancy phenotypes, we estimated heritability using Genome-wide Complex Trait Analysis software (GCTA) [25]. Similar to GEMMA, this software estimates a relatedness matrix based on the pairwise genetic covariance. We estimated h_g_^2^, the amount of variance in the trait that was explained by the interrogated SNPs. For h_g_^2^, GCTA fits the given phenotype with a linear mixed model, while it uses the estimated relatedness matrix as the variance term, to estimate the variance explained by all SNPs.

## Results

The characteristics of the 1,686 women included in this analysis are summarized in **Table 1.** They averaged 34.7 years in age and a body mass index of 20.1. All women in the study population were married. The average number of pregnancies and children per woman was 3.1 (slightly higher than the reported national average for rural women). 438 (26.0%) women reported use of hormonal contraceptives.

**Table 1:** Characteristics of the 1,686 women included in GWAS analyses.

Phenotype	N (%)
**Age mean (sd)**	34.7 (9.6)
**BMI mean (sd)**	20.1 (3.2)
**TV ownership**	980 (58.1)
**Contraceptive use**	438 (26.0)
**Urinary arsenic (μg/g-creatnine) mean (sd)**	276.5 (257.8)
**Ever Regular Smoker**	138 (8.2)
**Number of Children**	
0	41 (2.4)
1	121 (7.2)
2	275 (16.3)
3	313 (18.6)
4	256 (15.2)
5	230 (13.6)
6	179 (10.6)
7	98 (5.8)
8	83 (4.9)
9	43 (2.6)
10+	47 (2.8)
**Number of Pregnancies**	
0	42 (2.5)
1	122 (7.2)
2	275 (16.3)
3	318 (18.9)
4	254 (15.1)
5	229 (13.6)
6	182 (10.8)
7	92 (5.5)
8	84 (5.0)
9	44 (2.6)
10+	44 (2.6)

### Genome-Wide Association Analysis

**Fig 1:**
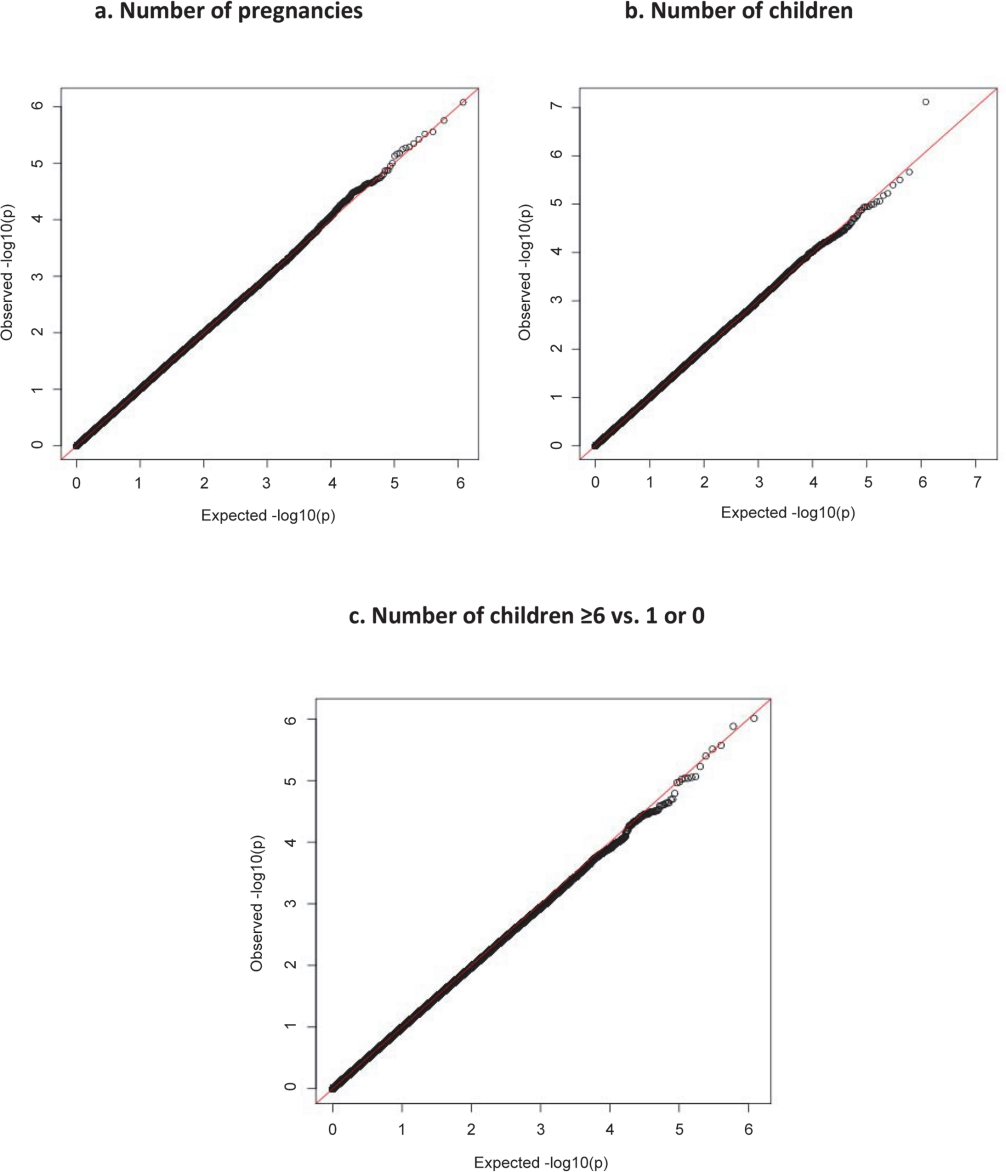
Quantile-quantile (Q-Q) plot for number of pregnancies (a) number of children (b) and ≥6 vs. ≤1 children for overall study population (N = 1,686).

The quantile-quantile (Q-Q) plots showing the distribution of p-values for number of pregnancies and number of children in the overall study population (including hormonal contraceptive users) is presented in **Fig. 1A**. The observed p-values for number of pregnancies matched the expected values over the range of 1.0<-log10(P)<4.0. The very slight departure was observed at the extreme tail (-log10(P)>4.5) of the distribution of test statistics for number of pregnancies, suggesting that the associations identified are likely due to true variants rather than potential biases such as genotyping error, sample relatedness, or potential population stratification. Similar findings were observed for the Q-Q plot of number of children and extreme number of children (**Fig. 1B and 1C**) where the observed p-values for number of children matched the expected values over the range of 1.0<-log10(p)<4.0 and the departure was observed at the extreme tail (-log10(P)>4.5) of the distribution of test statistics.

**Fig 2:**
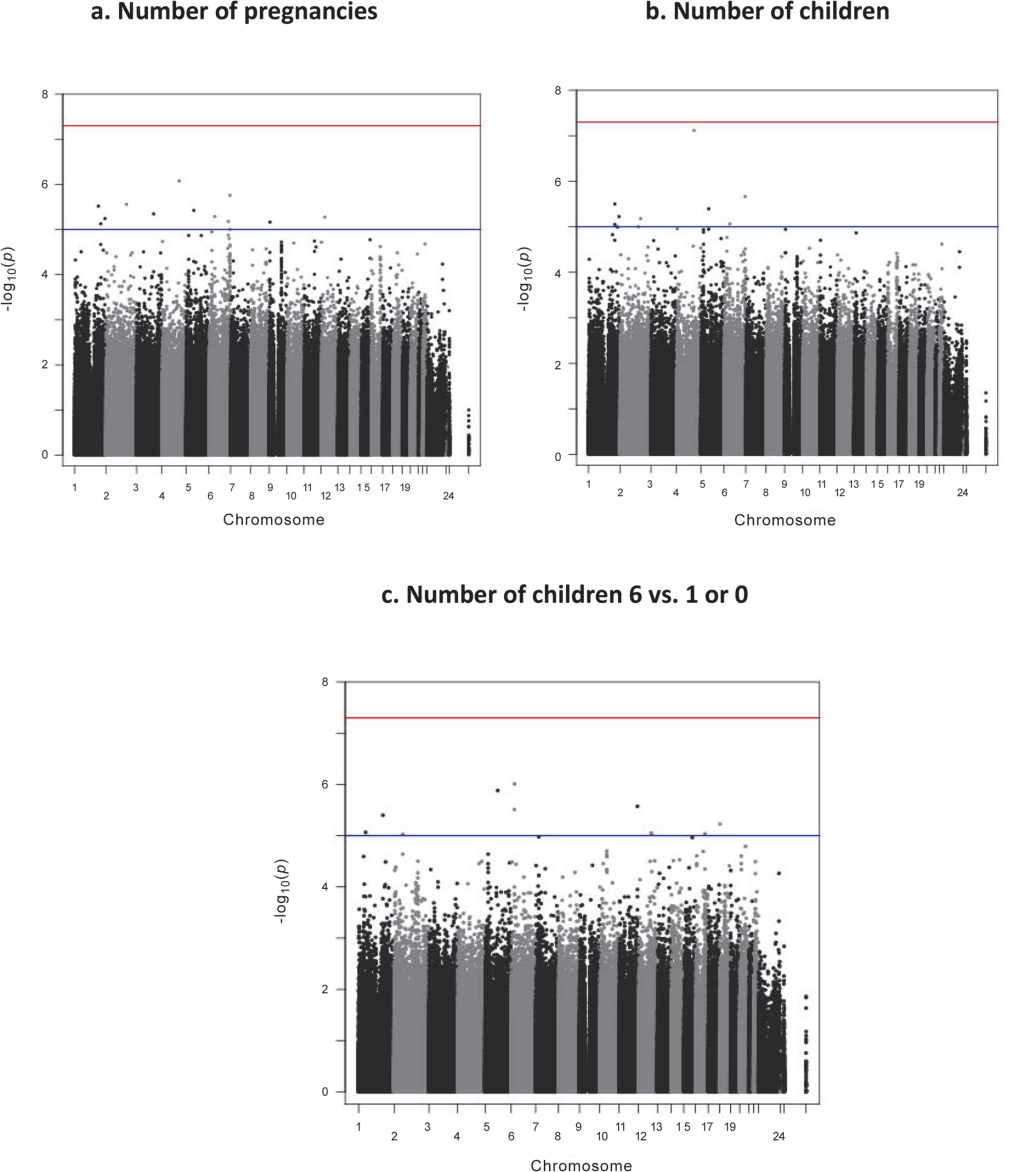
Genome-wide SNP association signals for number of pregnancies (a) number of children (b) and ≥6 vs. ≤1 children (c) for overall study population (N = 1,686).

**Fig 3:**
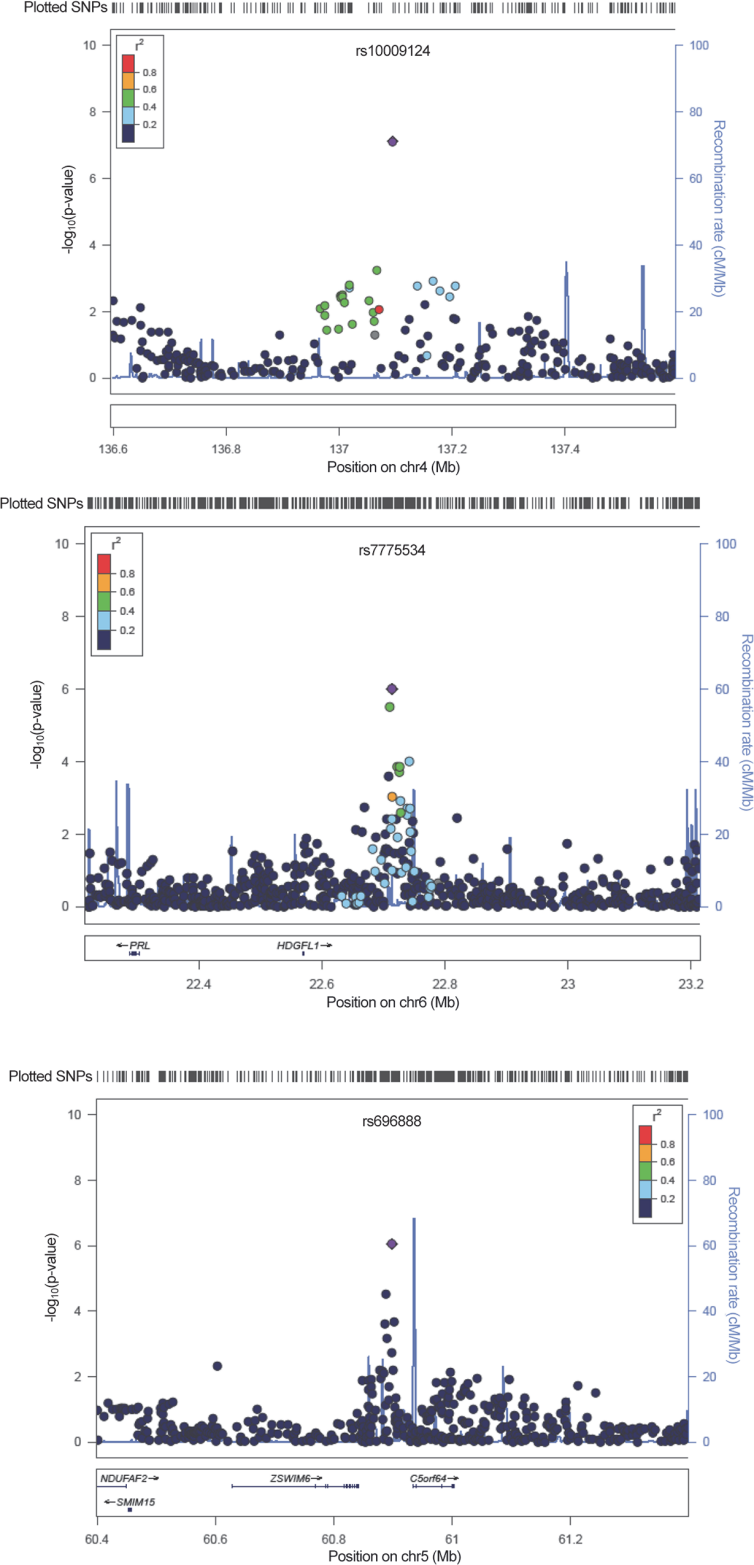
Locuszoom plots of SNPs rs10009124, rs7775534, and rs696888.

Although none of the SNPs reached the Bonferroni corrected significance level of 5 X10^–8^, 13 SNPs on chromosomes 1, 2, 3, 4, 5, 6, 9, and 12 reached P<10^–5^ for number of pregnancies (**Fig. 2A**) and nine SNPs on chromosomes 1, 2, 4, 5, and 6 were the most strongly associated SNPs at a significance level of P <10^–5^ for number of children (**Fig. 2B**). The most significant association was detected at rs100009124 for both number of pregnancies (P = 8.40E-07) and number of children (P = 7.65E-08) (**Table 2**). It is located in a gene desert (**Fig. 3**). In the GWAS analysis of extreme numbers of children (≥6 vs. ≤1), eleven SNPs on chromosomes 1, 2, 5, 6, 11, 12, and 18 reached the genome-wide threshold level of <10^–5^ for number of children (**Fig. 2C**). The top SNP was located on chromosome 6, near the gene *HDGFL1*, a hepatoma-derived growth factor (**Fig. 3**).

**Table 2:** SNPs with number of pregnancies number of children (linear) or extreme number children (≥6 compared to ≤1) in Bangladeshi women (p<1X10–5) for entire study population and excluding hormonal contraceptive users.

SNPs	Beta	P-value	Chromosome	BP	SNPs	Beta	P-value	Chromosome	BP
All participants (N = 1,686)					Excluding HC users (N = 1,248)				
**Number of Children**									
rs10009124	0.31	7.65E-08	4	137314199	rs696888	-0.46	5.24E-07	5	60933914
rs1105228	0.29	2.16E-06	6	165626463	rs10009124	0.36	5.54E-07	4	137314199
rs12408989	-0.38	3.15E-06	1	207074401	rs1105228	0.37	1.53E-06	6	165626463
rs696888	-0.34	4.03E-06	5	60933914	rs6464448	-0.69	2.65E-06	7	139259541
rs16841877	-0.34	5.97E-06	1	239840909	rs253185	-0.44	3.25E-06	5	60924067
rs4295021	0.28	6.63E-06	2	162540002	rs16841877	-0.43	3.45E-06	1	239840909
rs16872971	1.90	8.64E-06	6	45036931	rs1978180	-0.67	4.06E-06	7	139255786
rs12406463	-0.36	8.93E-06	1	207068843	rs4295021	0.35	4.07E-06	2	162540002
rs13025486	-0.52	1.00E-05	2	143728969	rs2575372	-0.36	4.72E-06	16	1755758
					rs3801153	-0.68	5.18E-06	7	139234645
					rs7919841	-0.34	6.64E-06	10	3829642
					rs7036692	-0.36	6.85E-06	9	5962586
					rs7136873	0.77	7.12E-06	12	2741947
					rs17809806	-0.44	9.96E-06	12	10723410
**Number of Pregnancies**									
rs10009124	0.27	8.40E-07	4	137314199	rs696888	-0.42	8.88E-07	5	60933914
rs1105228	0.28	1.76E-06	6	165626463	rs1105228	0.34	1.32E-06	6	165626463
rs4295021	0.27	2.79E-06	2	162540002	rs4295021	0.33	1.62E-06	2	162540002
rs12408261	-0.28	3.05E-06	1	187679798	rs16839793	-0.35	2.33E-06	3	134105347
rs696888	-0.32	3.79E-06	5	60933914	rs7136873	0.77	2.38E-06	12	2741947
rs16839793	-0.28	4.52E-06	3	134105347	rs4146401	0.39	3.16E-06	5	120666583
rs16872971	1.87	5.18E-06	6	45036931	rs12408261	-0.33	3.37E-06	1	187679798
rs10843287	-0.26	5.35E-06	12	29010043	rs2575372	-0.34	3.70E-06	16	1755758
rs16841877	-0.33	5.71E-06	1	239840909	rs11716468	0.32	4.12E-06	3	194557227
rs2813490	-0.27	6.63E-06	6	152510077	rs16841877	-0.40	4.58E-06	1	239840909
rs7869190	-0.32	6.88E-06	9	5541945	rs7869190	-0.39	4.83E-06	9	5541945
rs12408989	-0.34	7.47E-06	1	207074401	rs10009124	0.31	4.92E-06	4	137314199
rs510579	0.25	1.00E-05	6	165633951	rs16872971	2.25	5.56E-06	6	45036931
					rs510579	0.31	5.57E-06	6	165633951
					rs4687410	0.31	6.68E-06	3	194560209
					rs9325747	-0.43	6.69E-06	8	15112351
					rs11020998	0.31	7.34E-06	11	94361182
					rs561155	0.31	7.62E-06	6	165635865
**Number of Children 6+ vs. 0 or 1**										
rs7775534	0.08	9.76E-07	6	22821931	rs6491036	0.26	3.63E-06	13	24770402
rs1429606	0.23	1.31E-06	5	87412858	rs10505172	-0.12	5.64E-06	8	113712464
rs1808470	0.10	2.68E-06	11	127251725	rs6864602	0.66	6.13E-06	5	144612064
rs4712710	0.08	3.10E-06	6	22818020					
rs28588043	0.16	4.01E-06	1	169232636					
rs7239865	0.08	5.93E-06	18	1961193					
rs186478	0.21	8.62E-06	1	48949737					
rs1368013	-0.08	8.87E-06	12	89301022					
rs1368011	-0.08	9.20E-06	12	89312272					
rs35320143	-0.09	9.21E-06	16	65195746					
rs12991146	0.26	9.42E-06	2	59964726					

All participant models were adjusted for age, study stage, TV ownership, hormonal contraceptive use, enrollment year, and relatedness. The models excluding HC users were adjusted for age, study stage, TV ownership, enrollment year, and relatedness; BP- base pair.

We also present our top SNPs in Table 2 excluding hormonal contraceptive users. The most strongly associated SNP for both number of children (P = 5.24E-07) and number of pregnancies (P = 8.88E-07) was rs696888. Rs696888 is located near *C5orf64*, an open reading frame, and *ZSWIM6*, a zinc ion binding gene (**Fig. 3**). The top SNP identified in the overall study population (rs100009124) has the second strongest association for number of children (5.24E-07) and reached P<10^–5^ for number of pregnancies (**Table 2**).

### Heritability

We estimated heritability for the parity traits by estimating h_g_^2^, which is the upper limit of the amount of phenotypic variance we could have expected to explain with our GWAS. The estimated the heritability of these phenotypes from our genotype data for number of children was h_g_^2^ = 0.149 (SE = 0.24, p-value = 0.265) and the estimated heritability for number of pregnancies was h_g_^2^ = 0.007 (SE = 0.22, p-value = 0.487). We subsequently did not find evidence that the SNPs interrogated by our genotyping and imputation were associated with the phenotypic variation in the continuous parity-related traits of this study population.

## Discussion

In one of the first investigations of this hypothesis using a GWAS approach, we identified a borderline significant association for rs100009124 and number of children when corrected for multiple comparisons in our analysis. The same SNP was the top (but non-significant) association for number of pregnancies. This SNP appears to be in a gene desert on chromosome 4. When we excluded hormonal contraceptive users, the association with rs100009124 remained a top (but non-significant) association for number of children and number of pregnancies, but the strongest association for both the number of children and number of pregnancies was in rs696888, a SNP on chromosome 5 located near *C5orf64* and *ZSWIM6*. In the analysis of extreme number of children, the SNP on chromosome 6 that was the top (but non-significant) association appears to be closest to the gene *HDGFL1*.

Consideration of the nearest gene and gene function for the top SNPs identified in this analysis was limited. The only borderline significant SNP identified in this project is located in a region without a gene within 1 Mb- a gene desert. However, there is non-coding RNA (LINC00613) within ∼100kb. To our knowledge, there is no previous report of this SNP in the fertility literature. For the analyses excluding hormonal contraception users, the genes of interest appeared to be *C5orf64* (an open reading frame) and *ZSWIM6* (a zinc ion binding gene). Neither of these genes has been indicated in the fertility literature previously. However, the gene near the SNP associated with extreme number of children is *HDGFL1*, a hepatoma-derived growth factor. This gene has been reported previously to be associated with mild intellectual disability in an investigation of the genetic factors in father-daughter incest [23].

At present, there are a modest number of mutations, particularly in genes expressed in the hypothalamus, pituitary, gonads, and outflow tract are known to cause infertility in humans [24]. Most of these disrupt normal puberty and subsequently cause infertility. However, in our dataset, none of the known genes involved in fertility appeared to play a role. In GWAS investigation of fertility in animal studies, particularly in cows and bulls, investigators have reported top SNPs located close to or in the middle of genes with functions related to male fertility, such as the sperm acrosome reaction, chromatin remodeling during the spermatogenesis, and the meiotic process during male germ cell maturation [26–27]. However, we did not collect information on children or pregnancies for males in our study population and our findings are subsequently restricted to females.

This study was conducted in a cohort recruited originally to understand the health effects of arsenic. There is evidence in that arsenic exposure alters female reproductive physiology [28]. However, when we considered arsenic exposure in our parity models, we did not observe an effect. In addition, a limitation of this GWAS of parity is that information on known reproductive issues was not elicited. We also focus on our analysis on females as fertility information was not collected from males, but it is possible that male fertility impacts the ability to reproduce as well. The collection of additional fertility related data would enhance the suitability of the parity phenotypes reported here for GWAS analyses.

As investigation of this hypothesis is novel, the findings presented here offer some insight into genetic variation and parity. Although our null GWAS doesn’t rule out substantial heritability as it’s possible that many very weak effects could account for parity variation, overall, our findings are consistent with a low heritability of phenotypic variability for parity. In summary, our genome-wide association study of number of pregnancies and number of children in Bangladesh did not confer strong evidence of common variants for parity variation. However, our results suggest that future studies may want to consider the role of specific SNPs on chromosomes 4, 5 and 6 in their analysis.

## References

[1] Fidler AT, Bernstein JJ (1999) Infertility: from a personal to a public health problem, Public Health Rep 114:494–511. 10670617 10.1093/phr/114.6.494 PMC1308532

[2] Mitra SN, Ali MN, Islam S, Cross AR, Saha T (1994) Bangladesh Demographic and Health Survey 1993–94. Calverton, Maryland: National Institute for Population Research and Training (NIPORT),Mitra and Associates, and Macro International Inc.

[3] Mitra SN, Al-Sabir A, Cross AR, Jamil K (1997) Bangladesh Demographic and Health Survey 1996–97. Dhaka, Bangladesh and Calverton, Maryland [USA]: National Institute for Population Research and Training (NIPORT), Mitra and Associates, and Macro International Inc.

[4] National Institute of Population Research and Training (NIPORT), Mitra and Associates, ORC Macro (2005). Bangladesh Demographic and Health Survey 2004. Dhaka, Bangladesh and Calverton, Maryland [USA]: National Institute of Population Research and Training, Mitra and Associates, and ORC Macro.

[5] National Institute of Population Research and Training (NIPORT), Mitra and Associates, Macro International (2009) Bangladesh Demographic and Health Survey 2007. Dhaka, Bangladesh and Calverton, Maryland, USA:National Institute of Population Research and Training, Mitra and Associates, and Macro International.

[6] International Centre for Diarrhoeal Disease Research, Bangladesh (ICDDR,B). (2002) Health and demographic surveillance system—Matlab: Registration of health and demographic events 2000 Vol. 33 Dhaka, Bangladesh: ICDDR,B.

[7] National Institute of Population Research and Training (NIPORT), Mitra and Associates, Macro International (2012) Bangladesh Demographic and Health Survey 2011. Dhaka, Bangladesh and Calverton, Maryland, USA:National Institute of Population Research and Training, Mitra and Associates, and Macro International.

[8] Kohler HP, Rodgers JL, Miller WB, Skytthe A, Christensen K (2006) Bio-social determinants of fertility. Int J Androl 29: 46–53. 16466523 10.1111/j.1365-2605.2005.00606.x

[9] Kosova G, Abney M, Ober C (2010) Colloquium papers: Heritability of reproductive fitness traits in a human population. Proc Natl Acad Sci U S A 107:: Suppl 11772–8. 10.1073/pnas.0906196106 PMC2868280 19822755

[10] Simoni M, Tempfer CB, Destenaves B, Fauser BC (2008) Functional genetic polymorphisms and female reproductive disorders: Part I: Polycystic ovary syndrome and ovarian response. Hum Reprod Update 14: 459–484. 10.1093/humupd/dmn024 18603647 PMC2515090

[11] Tempfer CB, Simoni M, Destenaves B, Fauser BC (2009) Functional genetic polymorphisms and female reproductive disorders: part II–endometriosis. Hum Reprod Update 15: 97–118. 10.1093/humupd/dmn040 18805939 PMC2639061

[12] O'Flynn, O'Brien KL, Varghese AC, Agarwal A (2010) The genetic causes of male factor infertility: a review. Fertil Steril 93: 1–12. 10.1016/j.fertnstert.2009.10.045 20103481

[13] Yoon SH, Choi YM, Hong MA, Lee GH, Kim JJ, et al (2010) Estrogen receptor {alpha} gene polymorphisms in patients with idiopathic premature ovarian failure. Hum Reprod 25: 283–287. 10.1093/humrep/dep375 19861327

[14] Krausz C, Giachini C (2007) Genetic risk factors in male infertility. Arch Androl 53: 125–133. 17612870 10.1080/01485010701271786

[15] Choi J, Koh E, Suzuki H, Maeda Y, Yoshida A, et al (2007) Alu sequence variants of the BPY2 gene in proven fertile and infertile men with Sertoli cell-only phenotype. Int J Urol 14: 431–435. 17511727 10.1111/j.1442-2042.2007.01741.x

[16] Matzuk MM, Lamb DJ (2008) The biology of infertility: research advances and clinical challenges. Nat Med 14: 1197–1213. Fisher RA (1958) The genetical theory of natural selection. New York: Dover Publications Inc. 10.1038/nm.f.1895 18989307 PMC3786590

[17] Ahsan H, Chen Y, Parvez F, Argos M, Hussain AI, et al (2006) Health Effects of Arsenic Longitudinal Study (HEALS): description of a multidisciplinary epidemiologic investigation. J Expo Sci Environ Epidemiol 16: 191–205. 16160703 10.1038/sj.jea.7500449

[18] Purcell S, Neale B, Todd-Brown K, Thomas L, Ferreira MA, et al (2007) PLINK: a tool set for whole-genome association and population-based linkage analyses. Am J Hum Genet 81: 559–575. 17701901 10.1086/519795 PMC1950838

[19] Pierce BL, Kibriya MG, Tong L, Jasmine F, Argos M, Roy S, et al (2012) Genome-wide association study identifies chromosome 10q24.32 variants associated with arsenic metabolism and toxicity phenotypes in Bangladesh.PLoS Genet. 8(2):e1002522 10.1371/journal.pgen.1002522 22383894 PMC3285587

[20] Kang HM, Sul JH, Service SK, Zaitlen NA, Kong SY, Freimer NB, et al (2010) Variance component model to account for sample structure in genome-wide association studies. Nat Genet. Apr;42(4):348–54. 10.1038/ng.548 20208533 PMC3092069

[21] Chen WM, Abecasis GR. Family-based association tests for genomewide association scans. (2007) Am J Hum Genet. Nov;81(5):913–26. 17924335 10.1086/521580 PMC2265659

[22] Hoggart CJ, Clark TG, De Iorio M, Whittaker JC, Balding DJ. (2008) Genome-wide significance for dense SNP and resequencing data. Genet Epidemiol. Feb;32(2):179–85. 10.1002/gepi.20292 18200594

[23] Ansermet F, Lespinasse J, Gimelli S, Béna F, Paoloni-Giacobino A. (2010) Mild intellectual disability associated with a progeny of father-daughter incest: genetic and environmental considerations. J Child Sex Abus. May;19(3):337–44. 10.1080/10538711003788991 20509080

[24] Layman LC. Genetic causes of human infertility. (2003) Endocrinol Metab Clin North Am. Sep;32(3):549–72. Review. 14575025 10.1016/s0889-8529(03)00040-9

[25] Yang J, Benyamin B, McEvoy BP, Gordon S, Henders AK, et al (2010) Common SNPs explain a large proportion of the heritability for human height. Nat Genet 42: 565–569. 10.1038/ng.608 20562875 PMC3232052

[26] Berry DP, Bastiaansen JW, Veerkamp RF, Wijga S, Wall E, Berglund B, et al (2012) Genome-wide associations for fertility traits in Holstein-Friesian dairy cows using data from experimental research herds in four European countries. Animal. Aug;6(8):1206–15. 10.1017/S1751731112000067 23217223

[27] Peñagaricano F, Weigel KA, Khatib H. (2012) Genome-wide association study identifies candidate markers for bull fertility in Holstein dairy cattle. Anim Genet. Jul;43 Suppl 1:65–71. 10.1111/j.1365-2052.2012.02350.x 22742504

[28] Golub MS, Macintosh MS, Baumrind N. (1998) Developmental and reproductive toxicity of inorganic arsenic: animal studies and human concerns. J Toxicol Environ Health B Crit Rev 1:199–237. 9644328 10.1080/10937409809524552

